# Precursor uptake assays and metabolic analyses in isolated tomato fruit chromoplasts

**DOI:** 10.1186/1746-4811-8-1

**Published:** 2012-01-13

**Authors:** Djédoux Maxime Angaman, Rocco Petrizzo, Francesc Hernández-Gras, Carmen Romero-Segura, Irene Pateraki, Montserrat Busquets, Albert Boronat

**Affiliations:** 1 Departament de Bioquímica i Biologia Molecular, Facultat de Biologia, Universitat de Barcelona, Avda. Diagonal 643, 08028-Barcelona, Spain; 2 Centre de Recerca en Agrigenòmica (CRAG), Consorci CSIC-IRTA-UAB-UB, Campus Universitat Auntònoma de Barcelona, Bellaterra-Cerdanyola del Vallès, 08193-Barcelona, Spain; 3 Unité Pédagogique et de Recherche (UPR) en Biochimie et Microbiologie, Unité Régionale de l'Enseignement Supérieur (URES) de Daloa, Université d'Abobo-Adjamé, 02 BP 150 Daloa, Côte d'Ivoire

**Keywords:** Carotenoid, chromoplast, tomato, lipid, ripening, uptake assay

## Abstract

**Background:**

Carotenoids are the most widespread group of pigments found in nature. In addition to their role in the physiology of the plant, carotenoids also have nutritional relevance as their incorporation in the human diet provides health benefits. In non-photosynthetic tissues, carotenoids are synthesized and stored in specialized plastids called chromoplasts. At present very little is known about the origin of the metabolic precursors and cofactors required to sustain the high rate of carotenoid biosynthesis in these plastids. Recent proteomic data have revealed a number of biochemical and metabolic processes potentially operating in fruit chromoplasts. However, considering that chloroplast to chromoplast differentiation is a very rapid process during fruit ripening, there is the possibility that some of the proteins identified in the proteomic analysis could represent remnants no longer having a functional role in chromoplasts. Therefore, experimental validation is necessary to prove whether these predicted processes are actually operative in chromoplasts.

**Results:**

A method has been established for high-yield purification of tomato fruit chromoplasts suitable for metabolic studies. Radiolabeled precursors were efficiently incorporated and further metabolized in isolated chromoplast. Analysis of labeled lipophilic compounds has revealed that lipid biosynthesis is a very efficient process in chromoplasts, while the relatively low incorporation levels found in carotenoids suggest that lipid production may represent a competing pathway for carotenoid biosynthesis. Malate and pyruvate are efficiently converted into acetyl-CoA, in agreement with the active operation of the malic enzyme and the pyruvate dehydrogenase complex in the chromoplast. Our results have also shown that isolated chromoplasts can actively sustain anabolic processes without the exogenous supply of ATP, thus suggesting that these organelles may generate this energetic cofactor in an autonomous way.

**Conclusions:**

We have set up a method for high yield purification of intact tomato fruit chromoplasts suitable for precursor uptake assays and metabolic analyses. Using targeted radiolabeled precursors we have been able to unravel novel biochemical and metabolic aspects related with carotenoid and lipid biosynthesis in tomato fruit chromoplasts. The reported chromoplast system could represent a valuable platform to address the validation and characterization of functional processes predicted from recent transcriptomic and proteomic data.

## Background

Chromoplasts are non-photosynthetic plastids specialized in the synthesis and accumulation of carotenoids, the most widespread group of pigments found in nature. Carotenoids provide distinctive coloration to flowers and fruits, important for the visual attraction of animals for pollination and seed dispersal. Carotenoids in plants are also precursors for the synthesis of the hormone abscisic acid and other apocarotenoids with biological activity [1]. They are also found in chloroplasts, where they play an essential role as accessory pigments for light harvesting, as stabilizers of the thylakoid membranes and as photoprotectors preventing photo-oxidative damage. Besides their interest as plant pigments, carotenoids have nutritional relevance as their incorporation in the diet provides health benefits, helping to prevent some types of cancer and vascular and degenerative diseases [2-4].

In most fruits, chromoplasts differentiate from chloroplasts at defined stages of the ripening process. Differentiated chromoplasts show high morphological diversity depending of the plant species and have been classified in different types according to their shape and suborganellar structures [5]. The conversion of chloroplasts into chromoplasts is distinguished by the breakdown of the photosynthetic apparatus and a massive synthesis and deposition of carotenoids [6]. Like in other non-green plastids, it is believed that chromoplasts depend on the external supply of metabolic precursors and on alternative sources of ATP and NADPH required for anabolic processes [7]. In particular, very little is known at present about the nature of the precursors and the processes responsible for providing the cofactors required to sustain carotenoid biosynthesis in chromoplasts [7]. Furthermore, the involvement of chromoplasts in other biochemical processes related to fruit ripening, like the production of volatiles, phytohormones or defence compounds remains largely unexplored.

Like the rest of isoprenoids, carotenoids are built from the 5-carbon precursors isopentenyl diphosphate (IPP) and dimethylallyl diphosphate (DMAPP). In plastids, IPP and DMAPP are synthesized from pyruvate and glyceraldehyde 3-phosphate (GAP) via the recently elucidated methylerythritol 4-phosphate (MEP) pathway [8]. Carotenoid biosynthesis requires an important expenditure of energy (ATP) and reducing power (NADPH). The origin of the pyruvate, GAP, ATP and NADPH required for carotenoid biosynthesis in non-photosynthetic tissues is currently unknown. It is likely, however, that the availability of these metabolic precursors and cofactors depends on import from the cytosol or on biochemical and metabolic processes operating in the chromoplasts that have not yet been characterized. In this respect, it is worth noting that recent transcriptomic and proteomic data have revealed a number of biochemical and metabolic processes potentially active in fruit chromoplasts [9-12] namely membrane transport systems, carbohydrate metabolism, amino acid metabolism, lipid biosynthesis and respiratory activities. Considering that chromoplast differentiation is a very rapid process, there is the possibility that some of the proteins identified in the proteomic analysis could represent remnant chloroplast proteins no longer having a functional role in chromoplasts. For instance, proteins involved in photosynthesis have been detected in the proteomic analysis of tomato fruit chromoplasts in spite that this organelle does not have photosynthetic activity [11]. Thus, identification of novel biochemical processes being predicted in chromoplasts has to be validated experimentally using appropriate experimental systems. As a first approach to identify and validate metabolic processes in chromoplasts, we have set up a method for high yield purification of intact and biologically active tomato fruit chromoplasts suitable for precursor uptake assays and metabolic analyses.

## Results and discussion

### Isolation and purification of tomato fruit chromoplasts

Although a variety of protocols has been reported for the isolation of chromoplasts from fruits and flowers of different plant species, there is no a general method that could be applied to all plant samples. This certainly reflects the high structural and functional diversity of chromoplasts found among plant species [5]. Surprisingly and in spite of the interest of tomato as a model system to study fruit ripening and carotenoid biosynthesis, only a few methods have been reported for the purification of tomato fruit chromoplasts [11,13-15]. Among them, the method described by Bathgate et al. [13] was considered as the most suitable one for metabolic studies considering the high purity of the isolated plastids as estimated by phase contrast and electron microscopy, the use of marker enzymes and RNA analysis. Furthermore, the purified chromoplast preparations were reported to be competent for *in vivo *labeling of proteins using exogenously supplied ^35^S-methionine [13]. However, in our hands this method gave very low yields, most likely as a consequence of strong organelle lysis during the first isolation steps. Consequently, we introduced some modifications aimed at improving this protocol. On the one hand, the composition of the homogenization and resuspension buffer was modified by using sorbitol instead of sucrose and the addition of polyvinylpolypirrolidone (PVPP), ascorbic acid and phenylmethylsulfonyl fluoride (PMSF). Also the removal of fruit skin prior to homogenization resulted in an easier homogenization of the tissue and the filtration of the homogenate was faster using a pre-filtration step through gauze before filtration through Miracloth. A detailed description of the modified protocol is given in the Methods section.

**Figure 1:**
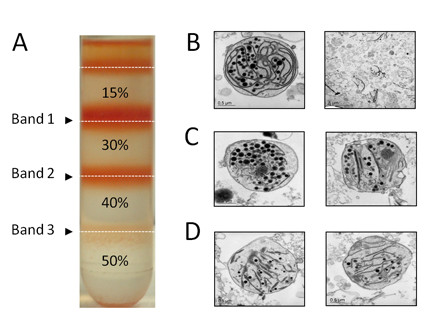
**Isolation of tomato fruit chromoplasts**. Chromoplasts isolated by centrifugation in a discontinuous sucrose density gradient (15, 30, 40 and 50%) (A). Electron micrographs of chromoplasts present in the 15-30% interface (B), 30-40% interface (C) and 40-50% interface (D). Similar electron microscopy images had been taken in two independent chromoplast preparations.

Figure 1A shows the typical pattern of bands obtained after sucrose density gradient centrifugation. Bands 1, 2 and 3 (corresponding to the 15/30%, 30/40% and 40/50% interfaces, respectively) were collected and chromoplasts recovered as indicated in the Methods section. The purity and intactness of the isolated chromoplasts were evaluated by transmission electron-microscopy. Samples from band 1 contained fully differentiated chromoplasts together with membranous and globular structures, likely derived from lysed chromoplasts (Figure 1B). Samples from band 2 contained developed chromoplasts harboring a large amount of globules but lower density of membranous structures than chromoplasts from band 1 (Figure 1C). Samples from band 3 contained poorly differentiated chromoplasts showing lower amount of globules and remnants of thylakoid structures (Figure 1D). A background of membranous-like structures can be observed in all the electron microscopy images shown in Figure 1. Similar structures were also present in the purified chromoplast preparations reported by Bathgate et al. [13]. The nature of these contaminating membranous-like structures is currently unknown.

To further assess the purity of the chromoplasts banding at the 30/40% interface, the activity of specific marker enzymes was determined and related to the total activity measured in the crude cell homogenate. Mitochondrial and peroxisomal contamination was excluded considering the very low level of succinate dehydrogenase and catalase activity (0.03% and 0.5% of the total activity present in the crude cell homogenate, respectively). The undetectable levels of alcohol dehydrogenase indicated that the chromoplast preparations were also free from cytosolic contamination. A typical isolation of tomato fruit chromoplasts starting from 300 g of pericarp tissue yielded ~1.8 × 10^8 ^chromoplasts, corresponding to about 500 mg of protein.

### Uptake assays using isolated tomato fruit chromoplasts

Since carotenoids represent the most relevant end product synthesized in the chromoplasts, we first studied the uptake of cytosolic intermediates potentially involved in the supply of the precursors needed for their biosynthesis. It is well known that the IPP and the DMAPP used for carotenoid biosynthesis in the plastids are synthesized by the MEP pathway. Although the origin of the pyruvate and the glyceraldehyde 3-phosphate used by 1-deoxy-D-xylulose 5-phosphate synthase, the first enzyme of the MEP pathway, is well established in chloroplasts, the metabolic origin of these metabolic precursors in non-photosynthetic plastids is currently unknown.

Studies using plastids isolated from sweet-pepper fruit and buttercup flowers revealed the presence of all the enzyme activities required for glycolytic conversion of glucose to pyruvate in both chloroplasts and chromoplasts [16,17]. However, the low activity levels of phosphoglycerate mutase and aldolase in chromoplasts let to suggest that the carbon flux to the lower part of glycolysis may be limited. Consequently, it was proposed that the provision of metabolites to the lower part of the glycolysis (e.g. pyruvate) could be maintained from the cytosol [16]. The operation of currently known relevant membrane transporters in non-green plastids [18-20] is in agreement with this proposal. Proteomic data recently reported by Barsan et al. [11] has revealed the presence of all glycolytic enzymes and the glucose translocator in tomato fruit chromoplasts, in agreement with the view that glucose could represent a likely precursor for carotenoid biosynthesis.

It is well documented that malate and citrate accumulate at similar levels during tomato fruit development. However, as fruit begins to ripen, malate levels start to decrease whereas those of citrate are still increasing [21-24]. Since malate can be converted into pyruvate by the action of the plastidic NADPH-dependent malic enzyme, this metabolite has been considered to represent an alternative source of both pyruvate and NADPH in the chromoplast. In this regard malate has been reported as an efficient precursor of anabolic processes in other non-green plastids [25,26].

**Figure 2:**
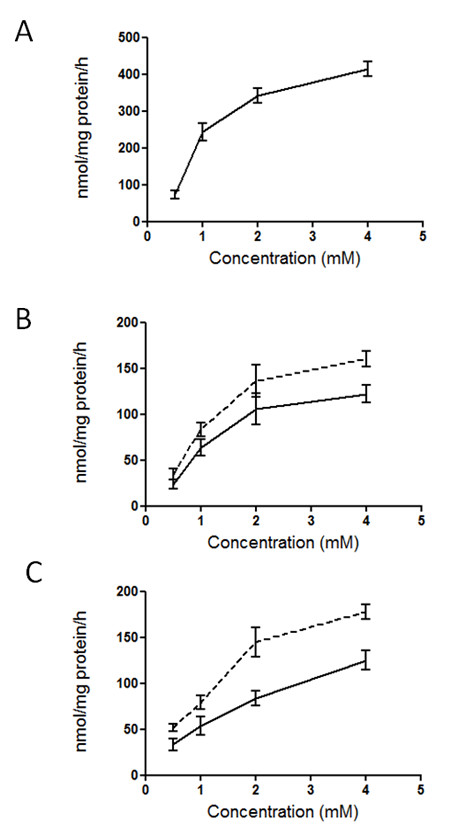
**Uptake of glucose, pyruvate and malate into isolated tomato fruit chromoplasts**. Chromoplasts were incubated in the presence of the indicated concentrations of glucose (A), pyruvate (B) and malate (C) containing 3.125 Bq of [^14^C]-glucose, [^14^C]-pyruvate and [^14^C]-malate, respectively. Dotted lines in graphs (B) and (C) correspond to chromoplasts preloaded with Na^+ ^and inorganic phosphate, respectively. Each value represents the mean and standard deviation of measures made with three separate preparations. Values are the mean +/- SE of duplicate measurements made with three independent chromoplast preparations.

As a first approach to identify precursors used for carotenoid biosynthesis in tomato fruit, we tested the ability of isolated chromoplasts to incorporate and metabolize a set of candidate radiolabeled metabolic intermediates. Thus we performed uptake assays using [^14^C]-glucose, [^14^C]-pyruvate or [^14^C]-malate. As shown in Figure 2 all these compounds were efficiently incorporated into chromoplasts, although glucose was incorporated at rates significantly higher than those of pyruvate and malate. In all cases, incorporation rates reached the highest values at precursor concentrations ranging close to 5 mM. The incorporation rates of [^14^C]-pyruvate and [^14^C]-malate were higher when chromoplasts were preloaded with sodium chloride and inorganic phosphate, respectively, in agreement with the involvement of known plastidial pyruvate and malate translocators [27-29].

### Metabolic studies using isolated tomato fruit chromoplasts

Since uptake assays measure both transport and metabolism of the precursors, the obtained results do not allow to conclude whether the differences in the incorporation rates actually reflect the operation of more efficient membrane transport systems or higher metabolic rates within the organelle (or a combination of both).

**Figure 3:**
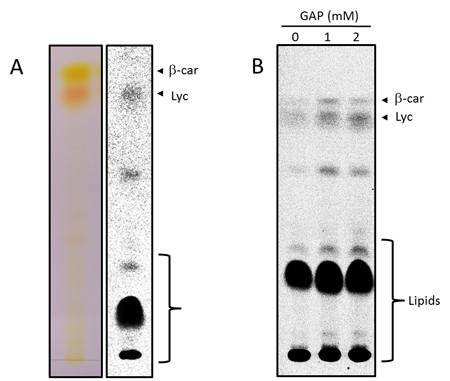
**Analysis of radiolabeled compounds in isolated tomato fruit chromoplasts**. Isolated chromoplasts were incubated with [^14^C]-pyruvate for 24 h and then extracted with hexane:acetone:methanol (2:1:1). Labeled compounds present in the organic fraction were separated by TLC and detected by autoradiography (A). Isolated chromoplasts were incubated with [^14^C]-pyruvate in the absence or presence of glyceraldehyde 3-phosphate (GAP) for 24 h and extracted with hexane:acetone:methanol (2:1:1). Labeled compounds present in the organic fraction were separated by TLC and detected by autoradiography (B). Eluents for TLC were hexane:ether:acetone (60:30:20) (A) and chloroform:methanol:water (65:25:4) (B). Results displayed are representative of at least three different experiments.

To evaluate the relative contribution of the metabolites tested above as precursors for carotenoid biosynthesis, chromoplasts were incubated with [^14^C]-glucose, [^14^C]-pyruvate and [^14^C]-malate under the same conditions used for uptake assays and at different times samples were withdrawn and extracted with a mixture of hexane:acetone:methanol (2:1:1, [v/v]). The labeled compounds present in the organic fraction were analyzed by TLC and autoradiography. To our surprise, and as exemplified in Figure 3A for [^14^C]-pyruvate, only a small fraction of the radioactivity (less than 5%) was incorporated into lycopene (the major carotenoid present in tomato fruit) and β-carotene. In contrast, most of the radioactivity was found in more polar compounds, which after TLC analysis using appropriate mobile phases were identified as a mixture of neutral and polar lipids (data not shown). A detailed analysis of the labeled lipids is currently underway.

The relatively high incorporation rate of the tested precursors into lipids has revealed that lipid biosynthesis is an active metabolic process in tomato fruit chromoplasts. However, the synthesis of fatty acids and complex lipids in chromoplasts is not an unprecedented observation as it was also reported in isolated daffodil petal chromoplasts [30]. In contrast, the low incorporation rates into lycopene and β-carotene was unexpected as it is widely accepted that carotenoid biosynthesis is a very active process during tomato fruit ripening. At present it is not known whether these results reflect the real situation in the ripening fruit. It is likely however that these results could reflect the lack of a particular metabolic intermediate or cofactor necessary for carotenoid biosynthesis not supplied in the incubation buffer used. In this respect, the availability of GAP could be considered as a potential limiting factor for carotenoid biosynthesis in our incorporation assays. On the one hand both GAP and pyruvate are the substrates of 1-deoxy-D-xylulose 5-phosphate synthase, the first enzyme of the MEP pathway. On the other hand, the reaction catalyzed by this enzyme is known to play a limiting role in carotenoid biosynthesis during tomato fruit ripening [31]. To evaluate this possibility, incorporation assays were performed using [^14^C]-pyruvate supplemented with different concentrations of GAP. As shown in Figure 3B, incorporation of radioactivity into lycopene was increased to some extent, suggesting a limiting pool of GAP in the isolated chromoplasts. The fact that the labeling of some lipids also increased in the presence of GAP may also reflect its limiting availability in lipid biosynthesis as well. Further work is needed to check whether the GAP required by the chromoplast can be supplied by the operation of an endogenous metabolic pathway or if it is imported from the cytosol. The latter possibility is supported by the identification of a triose-phosphate translocator as a component of the tomato chromoplast proteome [11].

It is widely accepted that the IPP and the DMAPP required for plant carotenoid biosynthesis is provided by the plastidial MEP pathway. In agreement with this, the pharmacological block of the MEP pathway in tomato fruits at mature green stage inhibits fruit ripening [32]. However, it is also well documented that cytosolic IPP synthesized by the mevalonate (MVA) pathway can contribute to the synthesis of plastidial isoprenoids in different plant species [33-36]. In the case of tomato there are reports showing the labeling of lycopene and β-carotene in tomato fruits fed with [^14^C]-mevalonic acid [37-39]. These results indicate that cytosolic IPP (or a derived prenyl diphosphate) produced from the MVA pathway may contribute to the synthesis of carotenoids during fruit ripening. This could explain, at least in part, the low incorporation rates of pyruvate and malate into carotenoids in isolated chromoplasts.

The results shown in Figure 3B revealed that the labeling of β-carotene was higher than expected considering the high lycopene to β-carotene ratio found in the ripe fruit, where lycopene accounts for about 90% of the total carotenoids. These results suggested that the rate of β-carotene synthesis in the isolated chromoplasts was higher than that in the pericarp tissue. A plausible explanation could be related with the particular differentiation stage of the chromoplasts used in this work (banding at the 30/40% interface). As indicated above they lack the complex membranous structures present in the chromoplasts banding at the 15/30% sucrose interface and therefore may not be fully differentiated. It has been reported that the rate of β-carotene synthesis changes during fruit ripening. Thus, while the accumulation rate of β-carotene is relatively high during the early stages of ripening, it drastically decreases at more advanced stages of ripening [40]. Thus, it is possible that the obtained results could reflect the particular metabolic situation of the chromoplast preparations used.

The high rate of lipid biosynthesis observed in the isolated chromoplast system opens the possibility that fatty acid biosynthesis could compete with carotenoid biosynthesis for the availability of common metabolic precursors (e.g. pyruvate) as well as cofactors (e.g. ATP and NADPH). It is likely however that the availability of precursors and cofactors in the fruit are properly balanced to allow both pathways to proceed in a coordinated way during ripening.

### Metabolism of [^14^C]-pyruvate and [^14^C]-malate in isolated chromoplasts

**Figure 4:**
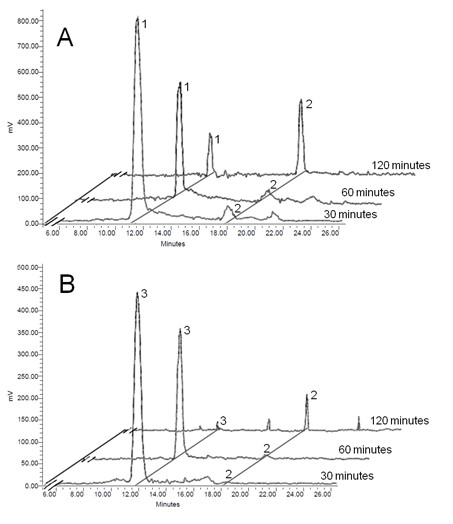
**Analysis of metabolic intermediates in isolated tomato fruit chromoplasts**. Isolated chomoplasts were incubated with [^14^C]-pyruvate (A) and [^14^C]-malate (B) for 30, 60 and 120 min. At the end of the incubation periods water soluble compounds were analyzed by HPLC (Aminex HPX-87H column) coupled to a radioactivity detector. Identified compounds are indicated: 1) pyruvate, 2) acetate and 3) malate. Results displayed are representative of at least three different experiments.

The high incorporation rates of [^14^C]-pyruvate and [^14^C]-malate into lipids suggested that metabolic channeling to acetyl-CoA (the biosynthetic precursor of fatty acids) was a very efficient process in isolated chromoplasts. These compounds share in common that they can readily be converted into acetyl-CoA through the operation of well know metabolic steps. Whereas malate can be converted into pyruvate in a reaction catalyzed by malic enzyme, the conversion of pyruvate into acetyl-CoA can be catalyzed by the pyruvate dehydrogenase complex. The presence of these enzymes in tomato fruit chromoplasts has been predicted from proteomic data [11]. To study the metabolism of these compounds in isolated chromoplasts we performed standard uptake assays using [^14^C]-pyruvate and [^14^C]-malate, and at different incubation times the [^14^C]-labeled metabolic intermediates were analyzed by HPLC. As shown in Figure 4, both [^14^C]-malate and [^14^C]-pyruvate were readily metabolized to [^14^C]-acetyl-CoA, thus confirming the presence of malic enzyme and pyruvate dehydrogenase activities in the isolated chromoplasts. The absence of [^14^C]-pyruvate in the incubations assays using [^14^C]-malate would suggest that the activity of pyruvate dehydrogenase activity is higher than that of malic enzyme in the chromoplast.

Acetyl-CoA can alternatively derive from acetate produced in mitochondria. The identification of acetyl-CoA synthase in the chromoplast proteome [11] opens the possibility that exogenous acetate could serve as an alternative source of acetyl-CoA used for lipid biosynthesis. Further experiments are needed to establish the relative contribution of pyruvate (and eventually acetate) as precursors for fatty acid biosynthesis in tomato fruit chromoplasts.

### Studies on cofactor requirements

The differentiation of fruit chloroplasts into chromoplasts correlates with a successive loss of the plastid autotrophic characteristics [5]. Fruit chloroplasts are photosynthetically active and able to synthesize ATP and NADPH, which can be used for CO_2 _fixation and starch biosynthesis [41]. During chromoplast differentiation chlorophylls are degraded and therefore the plastid becomes unable to photosynthetically produce ATP and NADPH. Concerning ATP, it has been proposed that an alternative source for the synthesis or uptake of this cofactor has to be induced in the chromoplast to sustain the biosynthetic capacity of this organelle [5,42]. In heterotrophic cells, most of the ATP synthesized in mitochondria is transported to cytosol and, from there, to different cell compartments (including plastids) through the action of specific nucleotide phosphate translocators [18].

**Figure 5:**
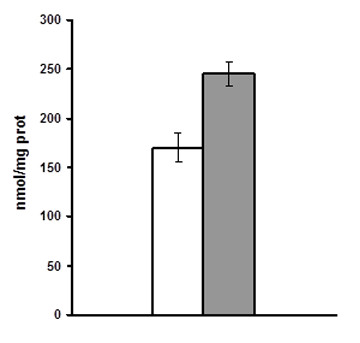
**Effect of ATP on lipid biosynthesis in isolated tomato fruit chromoplasts**. Incorporation of [^14^C]-pyruvate into lipids was measured in the absence (white bar) or presence (grey bar) of 5 mM ATP. Values are the mean +/- SE of measurements (in duplicate) made with two independent chromoplast preparations.

To check to what extent lipid biosynthesis in the chromoplast was dependent on the supply of external ATP incorporation, experiments were performed excluding ATP from the incubation buffer. As shown in Figure 5, lipid biosynthesis proceeded at a significantly high rate in the absence of exogenous ATP. Since lipid biosynthesis is a highly demanding energetic process, the obtained results indicate that tomato fruit chromoplasts could contain endogenous mechanisms to produce ATP. Alternative known possibilities are the phosphorylation at substrate level, the operation of an ATP synthase complex or metabolic recycling. Since several ATP synthase complex subunits have been detected in the chromoplast proteome [11], as well as several subunits from different respiratory complexes like NADPH dehydrogenase, it is possible that this organelle could retain the activity, at least in part, of the ATP synthase complex present in the chloroplast but driven by alternative electron donors not depending from the photosynthetic activity, similarly to what has been reported for chlororespiration [43]. Isolated chromoplasts could represent a suitable experimental tool to address these issues.

## Conclusions

We have set up a suitable system for metabolic and biochemical studies using isolated tomato fruit chromoplasts. The system is based on the development of a robust protocol for high-yield purification of intact and metabolically active chromoplasts from ripening fruits. Using targeted radiolabeled precursors we have been able to unravel novel aspects related with tomato fruit chromoplast metabolism. We have shown that a variety of metabolic precursors can be efficiently taken up by the isolated chromoplasts and metabolized through specific metabolic pathways. Labeling studies using [^14^C]-glucose, [^14^C]-pyruvate and [^14^C]-malate have revealed that lipid biosynthesis is a very active process in tomato fruit chromoplast and that metabolic channeling of metabolic precursors to acetyl-CoA is a very efficient process. We have also shown that tomato fruit chromoplasts could bear endogenous mechanisms to produce ATP used for anabolic processes. Furthermore, the isolated chromoplast system used in this work may represent an appropriate platform to address the validation and characterization of novel functional processes predicted in tomato fruit chromoplasts from recent transcriptomic and proteomic data.

## Methods

### Plant and fruit materials

Tomato (*Solanum lycopersicum *cv. Ailsa Craig) plants were grown under standard greenhouse conditions. Fruits were collected 8 to 12 days after breaking. Fruits were processed immediately after harvesting or stored at 4°C and used within the next 2-3 days.

### Chemicals

All chemicals used in this study were of the highest grade available and were purchased from Sigma-Aldrich, Amersham Biosciences (GE Healthcare) and Calbiochem. Radiochemicals were from American Radiolabeled Chemicals (Itisa Biomedica) and Amersham Biosciences (GE Healthcare).

### Isolation and purification of tomato chromoplasts

Chromoplasts were isolated using the method described by Bathgate et al. [13] with some modifications. Unless otherwise stated, all materials and solutions were kept at 2-4°C during the isolation and purification process. Fruits (about 300 g) were washed with 2.5% NaCl in distilled water for 15 min. About 200 g of pericarp tissue, after the removal of the skin, seeds and the gelatinous material of the locular cavities, was cut into small pieces with a razor blade and mixed with 2 volumes of buffer A (100 mM Tris-HCl pH 8.2, 0.33 M sorbitol, 2 mM MgCl_2_, 10 mM KCl, 8 mM EDTA, 10 mM ascorbic acid, 5 mM L-cysteine, 1 mM PMSF, 1% PVPP and 1 mM DTT). After homogenization with a Waring blender (three pulses at low speed) the homogenate was first filtered through 8 layers of gauze and then through 2 layers of Miracloth (Calbiochem). The debris retained in the gauze layers were recovered, mixed with one volume of buffer A and homogenized again. After filtration through gauze and Miracloth layers as described above, the homogenates were mixed and centrifuged for 2 min at 200 × g. The supernatant was recovered and centrifuged for 10 min at 5,000 × g. The obtained pellet was resuspended in 50 mL of buffer B (buffer A without PVPP) and centrifuged for 10 min at 5,000 × g. The pellet was resuspended in 4 mL of buffer B and chromoplasts were fractionated by ultracentrifugation on a discontinuous sucrose gradient (15%, 30%, 40% and 50% in Tris-HCl pH 7.4 supplemented with 1 mM DTT) for 1 h at 100,000 × g (Beckman SW 28 rotor). Chromoplast fractions banding at the 15-30%, 30-40% and 40-50% interfaces (Figure 1A) were recovered by gentle aspiration with a Pasteur pipette. The collected fractions were washed with one volume of buffer B and chromoplasts were recovered by centrifugation (10 min, 5,000 × g).

### Electron microscopy

Chromoplasts were washed, resuspended in 0.33 M sorbitol and fixed in a solution of 5% glutaraldehyde (475 mOms/Kg) in 0.1 M phosphate buffer (pH 7.4) at 4°C. After centrifugation for 2 min in a microfuge the pellet was fixed again in 2.5% glutaraldehyde, post-fixed in 1% osmium tetroxide (OsO_4_) containing potassium ferricianide (K_3_Fe(CN)_6_) (0.8%) in the same phosphate buffer for 1 h at 4°C, dehydrated in a graded acetone series and embedded in Spurr's resin. Blocks were polymerized for 48 h at 60°C. Thin sections (0.5-1 μm) were used to select the zone to explore at the electron microscope. Ultrathin sections (50-80 nm) were obtained with an ultramicrotome (Leica Ultracut UCT) using a diamond knife (Diatome). The ultrathin sections were mounted on copper grids and stained with 2% uranyl acetate for 10 min and with lead citrate for 30 min. Ultrastructural analysis was performed using a Jeol JEM 1010 operate at 80 Kv of acceleration with a Bioscan 812 camera (Gatan).

### Marker-enzyme assays

The activity of the enzymes succinate dehydrogenase, catalase and alcohol dehydrogenase was measured in purified chromoplast samples (resuspended in buffer B) and in crude fruit homogenate. Succinate dehydrogenase and catalase activities were measured in samples previously treated with 1% Triton X-100 for 10 min at 4°C. The assay's methods used for enzyme activities were those described in the literature: succinate dehydrogenase according to Graham [44], catalase according to Bergmeyer [45] and alcohol dehydrogenase according to Beaulieu et al. [46]. Marker-enzyme assay determinations were made in duplicate using two independent chromoplast preparations.

### Uptake assays of radiolabeled precursors

Uptake assays were performed using the double silicone oil layer centrifugation method described by Gross et al. [47] and modified by Weber et al. [48]. For malate and pyruvate uptake assays, aliquots of purified chromoplasts were preloaded with phosphate and Na^+ ^by incubation with 1 mM KH_2_PO_4 _and 0.1% NaCl, respectively, for 30 min on ice. After centrifugation at 4,000 × g for 4 min at room temperature, chromoplasts were resuspended in 100 μL of incorporation buffer (100 mM HEPES-KOH pH 7.6, 0.33 M sorbitol, 2 mM MnCl_2_, 10 mM MgCl_2_, 1 mM NADP, 1 mM NADPH, 5 mM ATP, 20 μM FAD) containing 0.5, 1, 2 and 4 mM of cold substrate (malate, pyruvate or glucose) supplemented with 3.125 Bq of the corresponding labeled substrate [U-^14^C]-glucose, ([(1,3), (2,4)-^14^C]-malate or [2-^14^C]-pyruvate, and incubated at 25°C. At the end of the specified incubation periods, chromoplast samples were loaded on top of a density gradient containing (from bottom to top) 100 μL of glycerol:methanol (2:1 [v/v]), 50 μL of silicone oil AR200 (Fluka, Deisenhofen, Germany), 50 μL of buffer B containing 220 mM sorbitol and 110 mM sucrose, 50 μL of silicone oil AR200 in a long polyethylene microcentrifuge tube of 0.4 mL (500-Q Quality Scientific Plastics) and then centrifugated at 14,000 × g during 30 s. Tubes were frozen in liquid nitrogen and the lower part containing the chromoplast pellet cut and placed into a vial containing 2 mL of scintillation liquid (Ecoscint H). The incorporated radioactivity was counted in a liquid scintillation analyzer (TRI-CARB 2100 TR, Packard).

### Analysis of radiolabeled compounds using TLC and autoradiography

After incubation with the radiolabeled precursors as indicated above, chromoplast samples were extracted with 1 mL of hexane:acetone:methanol (2:1:1, [v/v]) by shacking for 15 min. Distilled water (100 μL) was then added and vigorously mixed using a vortex. After centrifugation for 3 min at 5,000 × g, the organic fraction was transferred into a new tube. The extraction process was repeated. The organic solvent used for the two extractions was mixed and dried under a N_2 _gas flow. Samples were dissolved in 100 μL of chloroform and used for counting the incorporated radioactivity or for analysis by TLC (SIL G-25 UV 254 plates). Different mobile phases were used for the TLC analysis: hexane:toluene (7:3, [v/v]) for highly hydrophobic compounds, like carotenoids, and chloroform:methanol:water (65:25:4, [v/v]) for less hydrophobic lipids. After drying, the TLC plates were placed in contact with Fujifilm screens for bioimaging analyzer BAS-IPMS 2325 and scanned with Bioimager (Bio-Rad). Lipids were visualized by brief exposure to iodine vapor.

### Analysis of radiolabeled metabolic intermediates by HPLC

Radiolabeled metabolic intermediates were analyzed by HPLC using a Waters 600 HPLC system equipped with a Waters 2996 UV detector, a radioactivity detector and the Enpower software for data acquisition (Waters, USA). After incubation with the radiolabeled precursors as indicated above for different time periods chromoplasts samples (100 μL) were mixed with 100 μL of distilled water and vortexed for 1 min. Lysed chromoplast samples were centrifuged for 5 min at 14,000 × g and the supernatants were used for HPLC analysis. Stock solutions (100 mg/L) of the standards were dissolved in Milli-Q water. All solutions were filtered through a 0.45 μm membrane filter, and finally stored in a refrigerator before use. According to the experimental requirements, citric acid, oxaloacetic acid, succinic acid, fumaric acid, malic acid, acetic acid and pyruvic acid stock solutions were mixed in volume ratio of 1:10:5:10:5:15:10, and then diluted to required concentrations to prepare a serial mix of standard solutions. The mobile phase consisted on 0.27 mL of sulfuric acid diluted to 1 L with Milli-Q water, then filtrated through a 0.45 μm membrane filter and degassed before use. Metabolites profiling was performed using the ionic exchange Aminex HPX-87H column (7.8 mm i.d. × 300 mm, 9 μm) using 0.005 mol/L sulfuric acid as mobile phase at a flow rate of 0.5 mL/min. The column was maintained at 50°C. Injection volume was 10 μL for all runs. Target compounds were detected using a UV detector set at 210 nm and a radioactivity detector.

## List of abbreviations

HPLC: High-Performance Liquid Chromatography; TLC: Thin Layer Chromatography; MEP pathway: 2-C-methyl-D-erythritol 4-phosphate pathway; MVA pathway: mevalonate pathway; GAP: glyceraldehyde 3-phosphate; IPP: Isopentenyl diphosphate; DMAPP: dimethylallyl diphoshate.

## Competing interests

The authors declare that they have no competing interests.

## Authors' contributions

DMA and CRS performed the uptake assays and the metabolic analyses. RP and FHG participated in setting up the method for chromoplasts purification. IP and MB supervised experiments. AB supervised the experiments and prepared the manuscript. All authors read and approved the final manuscript.
